# International Rotifer Symposia: prospects and retrospects from Rotifera XI

**DOI:** 10.1186/1746-1448-2-6

**Published:** 2006-05-15

**Authors:** SSS Sarma

**Affiliations:** 1 Laboratory of Aquatic Zoology, UMF, Division of Research and Postgraduate Studies, National Autonomous University of Mexico, Campus Iztacala, Av. de los Barrios S/N, Los Reyes, AP 314, CP 54090, Tlalnepantla, State of Mexico, Mexico

## Abstract

The XI International Rotifer Symposium was held during 11–18 March, 2006 at the National Autonomous University of Mexico Campus Iztacala located at the North Mexico City (Mexico). These triennial international meetings, first organized in Austria by Late Ruttner-Kolisko in September 1976, are gradually becoming the focal point of discussion and collaboration from rotifer workers across the world. The present XI symposium was attended by 125 participants from 20 nations. During this meeting, different themes of rotifer research from morphology to molecular biology were considered. In addition, there were four invited lectures and four workshops covering different themes of the symposium. During the last 30 years, rotifer research has witnessed gradual shift from the conventional morphological taxonomy to molecular and evolutionary systematics. While the basic rotifer ecological studies continue today, applied areas such as ecotoxicology and aquaculture have taken key roles in the recent meetings. The international rotifer meetings provide ample opportunities not only for exchange of ideas and recent research, but also for material and in establishing inter-personal relationships. Over the last 30 years, the number of participants attending the rotifer meetings has increased.

## Background

Rotifers are small (<2 mm) and beautiful invertebrates, harmless to mankind. Their use as model organisms in teaching courses of biology, as a scandalous group in evolutionary ecology, as larval diet in aquaculture and as sensitive indicators of water quality has been widely recognized. Moreover, due to their high metabolic rates, they regenerate nutrients trapped in phytoplankton and detritus on which they feed [1]. There are about 2150 rotifer species globally. The fact that new species are currently discovered from different parts of the world suggests that the taxonomic studies on rotifers are far from complete in spite of its 300 year old history [2,3].

The tradition of triennial rotifer symposia was first initiated by the late Ruttner- Kolisko of Austria. This has had a stimulatory influence on rotifer research. So far 10 countries have hosted these symposia (Austria (twice), Belgium, Italy, Poland, Spain, Sweden, United Kingdom, USA and Thailand and Mexico). Unlike many other conferences, rotifer symposia are distinguished by keeping a permanent record of presentations through formal publication in the form of proceedings in a prestigious scientific journal. Though the proceedings of the first rotifer meeting appeared in *Archiv für Hydrobiologie*, the subsequent proceedings have taken an honourable place in *Hydrobiologia*. The proceedings of the XI Rotifer meeting are also expected to appear in the same journal (Table 1).

**Table 1:** Details of the International Rotifer Symposia so far held. Symp: Symposium series, Part.: number of participants; Nat.: Nations represented.

Symp	Date and place	Main organizer	Part. (no.)	Nat. (no.)	Journal, vol., year	No. of articles
I	21–26 Sept, 1976; Lunz, Austria	A Ruttner-Kolisko	38	15	Arch. Hydrobiol. Beih., 8, 1977,	52
II	17–21 Sept. 1979; Ghent, Belgium	HJ Dumont	51	16	Hydrobiologia, 73, 1980	42
III	30 Aug. – 4 Sept. 1982	B Pejler	70	22	Hydrobiologia, 104, 1983	52
IV	18–25 Aug. 1985	L May	68	23	Hydrobiologia, 147, 1987	50
V	12–17 Sept. 1988, Gargnano, Italy	C Ricci	83	20	Hydrobiologia, 186/187, 1989	52
VI	3–8 June 1991, Banyoles, Spain	MR Miracle	107	25	Hydrobiologia, 255/256, 1993	72
VII	6–11 June 1994, Mikolajki, Poland	J Ejsmont-Karabin	93	26	Hydrobiologia 313/314 1995	53
VIII	22–27 June 1997, Collegeville, Minn. USA	E Wurdak	97	22	Hydrobiologia, 387/388, 1998	64
IX	16–23 Jan 2000, Khon Kaen, Thailand	L-O Sanoamuang	117	26	Hydrobiologia 446/447, 2001	51
X	7–13 June 2003, Illmitz, Austria	A Herzig	113	28	Hydrobiologia 546, 2005	58
XI	11–18 March 2006, Mexico City, Mexico	SSS Sarma	125	20	Expected to appear in Hydrobiologia	in process

## Results and discussion

**Figure 1:**
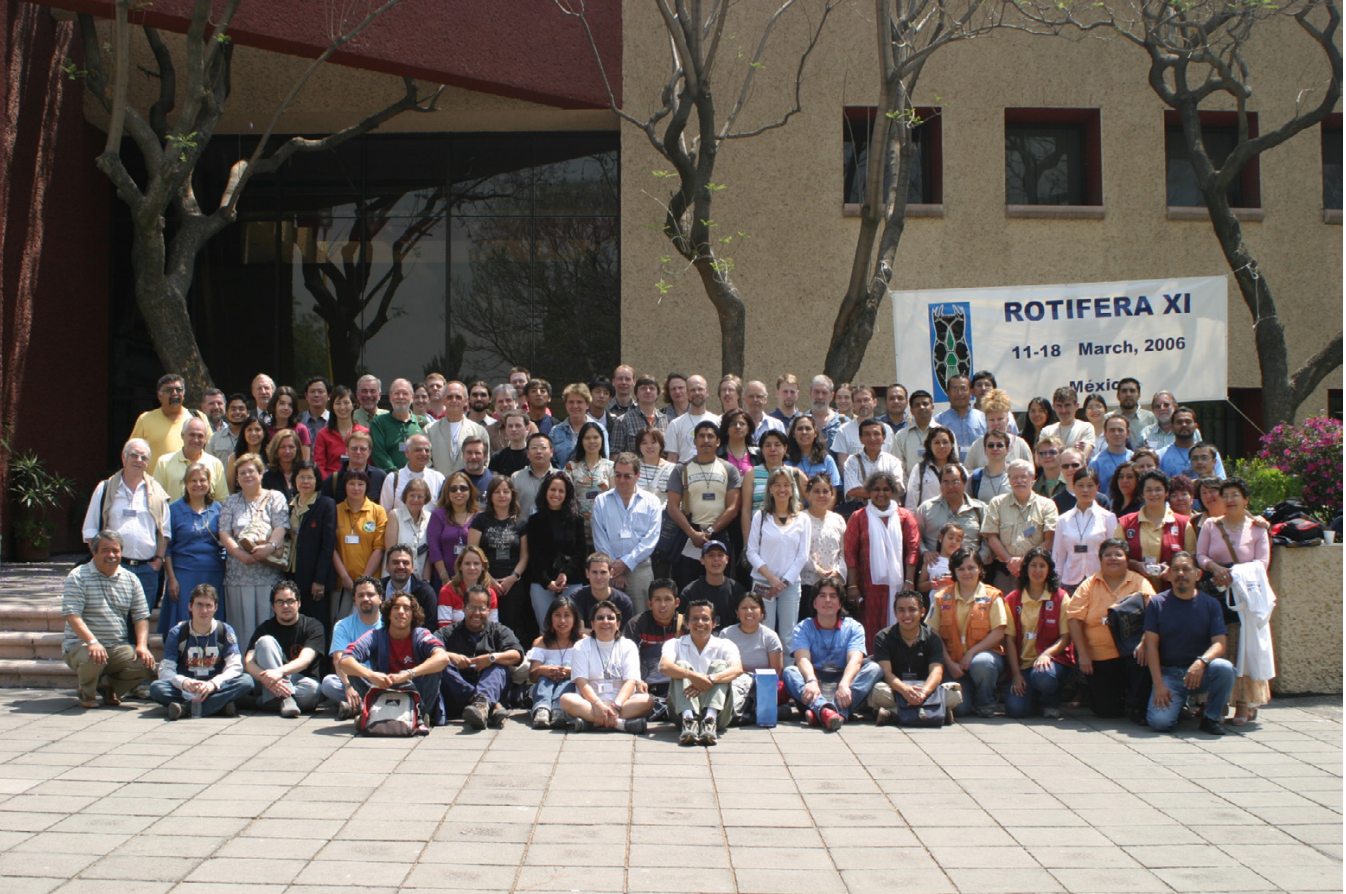
Participants of the XI International Rotifer Symposium held during March 11–18, 2006 at Mexico City (Mexico).

The XI international rotifer symposium was held during 11–18 March, 2006 at the National Autonomous University of Mexico Campus Iztacala located at the North Mexico City (Mexico). The abstracts of the meeting are available on the official website [4]. A total of 125 participants from 20 nations participated during this meeting (Figure 1). At the symposium five major areas were considered: 1. morphology, taxonomy, zoogeography and field ecology, 2. feeding, trophic interactions, behaviour, autecology and population ecology, 3. molecular biology, evolution, genetics and biochemistry, 4. aquaculture and mass production and 5. ecotoxicology and indicator species. In addition there were four invited lectures and four workshops covering different themes of the symposium. Workshops at the XI Rotifer Symposium were aimed at examining difficulties and/or problems and to uncover novel approaches that show promise in resolving these issues.

While many symposia are conducted at hotels, mainly due to logistic convenience, the present one was held in a university. In this way, several students and young faculty members were in fact stimulated by the presence of a great number of renowned researchers. This is evident from a report appeared recently in the University Gazette [5]. The Rotifera XI distinguished from all other previous ones in the sense that symposium took full advantage of the internet revolution. No formal invitation letters or the publicity material were printed on paper. All such information had been stored and mailed electronically. Two rotifer workers (Robert Wallace and Terry Snell) had attended all the 11 symposia without missing even a single meeting and thus received a university medal "Dedicated Rotifer Researcher". Five previous organizers of the rotifer meeting (HJ Dumont, MR Miracle, J Ejsmont-Karabin, LO Sanoamuang and A Herzig), who were present during Rotifera XI, also received the university medals for passing on the torch of successful rotifer symposium.

Rotifer symposia have a long and honourable tradition of a mid-conference excursion to some well known freshwater bodies. During the Rotifera XI the participants had an opportunity to visit a UNESCO world heritage site, the Xochimilco Lake.

### Emphasis on different themes

Presentations spanned several themes among which trophic interactions and laboratory experiments on the population and community ecology of rotifers constituted 45% of the total presentations (Table 2). The lowest input was on aquacultural applications of rotifers. In the section on morphology and taxonomy, brilliant electron microscopic techniques for preparing rotifer specimens were shown by Wilko Ahlrichs. G Melone and his colleagues presented information on water loss and the associated morphological changes during desiccation in bdelloids. They believe that the reduced body size of a desiccated bdelloid rotifer is primarily due to elimination of body cavities. Data on the zoogeographical distribution of rotifers from different continents were also presented by some participants. Scott Mills discussed the future of rotifer taxonomy mainly based on the euryhaline *Brachionus plicatilis *complex. New species descriptions have been largely restricted to *Brachionus *(from Mexico) and *Cephalodella *(North-West Germany) and *Dicranophorus *(Belgium). Based on an ongoing project, Elizabeth Walsh and her colleagues (in USA and the counterpart in Mexico) presented some preliminary data on rotifer diversity in the Chihuahua desert springs.

**Table 2:** Theme-wise contributions presented during the XI International Rotifer Symposium

Theme	Number of presentations
Feeding, trophic interactions, behaviour, autecology and population ecology	46
Morphology, taxonomy, zoogeography & field ecology	26
Molecular biology, evolution, genetics and biochemistry	16
Ecotoxicology and indicator species	13
Aquaculture and mass production	4

The section on the trophic interactions and population ecology considered many diverse aspects. The most interesting from the point of salinity stress and the rotifer ecology was the invited talk by Peter Starkweather. Though many rotifer species are restricted to freshwaters, some do inhabit hypersaline desert endorheic water bodies. Salinity has strong effects on both density and diversity of rotifers. S. Nandini and her colleagues presented data on rotifer diversity and abundance through seasons and at different depths from one of largest drinking water reservoirs in Mexico (Valle de Bravo).

The importance of morphological and molecular data on the rotifer phylogeny was considered during this symposium. This was supplemented by the workshop on DNA barcodes by C.W. Birky Jr. Cross mating experiments within the different geographical populations appear to show the existence of a species complex in the rotifer *Ephiphanes senta*. The limited molecular work on *Plationus *did not consider other biological characteristics such as male or resting egg morphology, demography or cross mating or mate-recognition factors for deriving phylogenetic relationship within Brachionidae. Hence its taxonomic status still remains unresolved. Mark Welch presented a nice account on phylogenetics and ribosomal gene evolution in the bdelloid rotifers, a group where males are totally absent.

The aquacultural application of rotifers is not steadily increasing with the same pace as shrimp or fish production, where they are widely used as live food. Some reasons seem to be: a) mass culture techniques of rotifers are nearly stabilized on a global scale [6] and possibly, since *Artemia *cysts are relatively easy to procure, marine aquaculture largely employs these anostracans. Nevertheless, attempts to improve the nutritional quality of rotifers were presented during this symposium.

Ecotoxicological aspects of rotifers were represented by as many as seven different groups. Most of these contributions during this symposium considered population level effects of different toxic substances on monogonont rotifers. Field-based studies were poorly represented. The workshop on ecotoxicology co-chaired by Terry Snell and Célia Joaquim-Justo invited lively discussion from the participants. There is a great practical application of rotifers in ecotoxicological evaluations and developing safety standards for water quality.

## Conclusion

Over the last 30 years, the number of participants attending the rotifer meetings is on the rise. The number of countries representing these events has stabilized to around 20. During the Rotifera XI, almost every aspect of rotifer research – both basic and applied was discussed.

## Competing interests

The author(s) declare that they have no competing interests.

## Authors' contributions

SSSS: Idea, design, data collection, interpretation and write up.
